# Supplementary material for: Chromothripsis during telomere crisis is independent of NHEJ, and consistent with a replicative origin
Source: Genome Res. 2019 May;29(5):737–49. doi: 10.1101/gr.240705.118 (PMC6499312; doi:10.1101/gr.240705.118)
Supplement: Supplemental Material [file supp_gr.240705.118_Supplemental_file_1.zip › contigs/annotated_contigs/DB78/contig.2.DB78_length_382_mean_cov_7.48691099476.docx]

**DB78_length_382_mean_cov_7.48691099476**

```
GGGTGGAAGGGCCGCAGGATAAGGAGGACCCCGGGGGGGGGAAAGGGGGAGGGAGGGAAGGTAAGCCAGGGAAGG|GGGGGGGGACCTG
                                                                            >chr6:159126
GGGGCAGGGTAACAGTGAGATCTGGAAAGTCACATAGAGCAGGAGGGACTCGGGGCAGGGTGACATTGAGGGCTGTAAGGTCACATAGA
596-159126774 + E=2e-76 p=0e+00
CCTGGGGACAGGGTGAC|AGGGCTGTAAGGTCACATAGACCTGGGGACAGGGTGACAGTGAGGGCTGTAAGG|TCACATAGACCTGGGG
                  >chr6:159126677-159126865 + E=9e-102
ACAGGGTGATAGGTGAGGGCTGTAAGGCCACGTAGGGCATGGGGGACCCAGGAACAGGGTGACAGTGATGGCTGTAAGGCCACATAGGG

CAGGGGGATCCAGAGACAGGAGCACCCTG
```
